# Retraction: Down-regulation of Rab10 inhibits hypoxia-induced invasion and EMT in thyroid cancer cells by targeting HIF-1α through the PI3K/Akt pathway

**DOI:** 10.1039/d1ra90027g

**Published:** 2021-01-21

**Authors:** Laura Fisher

**Affiliations:** Royal Society of Chemistry Thomas Graham House, Science Park, Milton Road Cambridge CB4 0WF UK advances-rsc@rsc.org

## Abstract

Retraction of ‘Down-regulation of Rab10 inhibits hypoxia-induced invasion and EMT in thyroid cancer cells by targeting HIF-1α through the PI3K/Akt pathway’ by Zhenyu Zhou *et al.*, *RSC Adv.*, 2018, **8**, 31682–31689, DOI: 10.1039/C8RA05855E

The Royal Society of Chemistry hereby wholly retracts this *RSC Advances* article due to concerns with the reliability of the data. The images in the article, and the raw data provided by the authors, were screened by an image integrity expert. The backgrounds to all the blots in the raw data are identical but the bands differ, and therefore, the bands have been pasted onto false backgrounds. The raw data, therefore, cannot be used to validate the published data.

Given the significance of the concerns about the validity of both the data in the article and the raw data provided by the authors, the findings presented in this paper are not reliable.

The authors have been informed but have not responded to any correspondence regarding the retraction.

Signed: Laura Fisher, Executive Editor, *RSC Advances*

Date: 7^th^ January 2021

## Supplementary Material

